# The Pituitary Immune Environment and Immunotherapy: From Hypophysitis to Pituitary Neuroendocrine Tumors

**DOI:** 10.3390/cells14181450

**Published:** 2025-09-16

**Authors:** Toru Tateno, Mariam Shahidi, Jian-Qiang Lu, Constance Chik

**Affiliations:** 1 Division of Endocrinology and Metabolism, Department of Medicine, University of Alberta, Edmonton, AB T6G 2G3, Canada; mshahidi@ualberta.ca; 2 Department of Pathology and Molecular Medicine, McMaster University, Hamilton, ON L8S 4K1, Canada; luj85@mcmaster.ca

**Keywords:** hypophysitis, immune checkpoint inhibitors, immune microenvironment, programmed cell death ligand 1, aggressive pituitary neuroendocrine tumors, immunotherapy

## Abstract

The immune landscape plays an important role in various pituitary diseases, ranging from hypophysitis to pituitary neuroendocrine tumors. Moreover, the use of immune checkpoint inhibitors (ICIs) has dramatically altered the landscape of cancer treatment by improving prognosis and overall survival in a multitude of advanced-staged malignancies, though their use in pituitary neuroendocrine tumors has remained limited. In this review, we will focus on selected topics to highlight the impact of the immune microenvironment on the function of the pituitary gland, namely, animal models of autoimmune hypophysitis, including ICI-induced hypophysitis as a common adverse event, and the importance of its early recognition in patients treated with ICIs. Using a case, we will provide an overview on the epidemiology, pathogenesis, clinical spectrum, diagnosis, predictors, and management of ICI-induced hypophysitis. We will also summarize the role of the immune microenvironment in pituitary neuroendocrine tumors with programmed cell death ligand 1 as a biomarker for treatment. Lastly, we will review the role of ICIs in the management of 40 patients with aggressive and metastatic pituitary neuroendocrine tumors. Current knowledge gaps in these topics will also be highlighted.

## 1. Introduction

The immune microenvironment plays a crucial role in the behavior and progression of various tumors. Immune checkpoint inhibitor (ICI) therapy has dramatically altered the landscape of cancer treatment by improving patient survival. However, use of these agents has led to recognition of ICI-induced hypophysitis (IIH), an immune-related adverse event of ICI treatment, characterized by inflammation of the pituitary gland, for which early recognition and timely management are crucial. In this review, we will focus on selected topics that highlight the impact of the immune microenvironment on pituitary gland function by describing animal models of autoimmune hypophysitis, IIH, and the role of the immune microenvironment in pituitary neuroendocrine tumors (PitNETs), with programmed cell death ligand 1 (PDL-1) as a biomarker.

### ICIs and Their Mechanisms of Action

The use of ICIs has drastically altered the landscape of cancer treatment by improving prognosis and overall survival in a multitude of advanced-staged malignancies. Since the approval of ipilimumab by the Food and Drug Administration (FDA) in 2011 for the treatment of melanoma [1], a growing number of ICIs have been approved for an expanding number of metastatic malignancies, including non-small-cell lung cancer, renal cell carcinoma, prostate cancer, Hodgkin’s lymphoma, and many more [2].

As summarized in a recent review, 12 ICIs had been approved by the FDA prior to 2024. These agents belong to four separate classes, including cytotoxic T-lymphocyte-associated antigen 4 (CTLA-4) inhibitors (ipilimumab and tremelimumab), programmed cell death protein 1 (PD-1) inhibitors (nivolumab, pembrolizumab, cemiplimab, dostarlimab, toripalimab, and retifanlimab), programmed cell death ligand 1 inhibitors (atezolizumab, durvalumab, and avelumab), and a lymphocyte–activating gene-3 (LAG-3) inhibitor (relatlimab) [3]. In 2024, two additional ICIs—cosibelimab and tislelizumab—were approved by the FDA [4,5].

ICIs are monoclonal antibodies that activate the immune system by blocking inhibitory interactions between immune checkpoints on T-cells and antigens on antigen-presenting cells (APCs) [6]. T-cell activation requires two sequential steps. In the initial priming phase, antigens are presented by the major histocompatibility complex (MHC) on APCs to the T-cell receptor (TCR) on T-cells. Following this, CD28, a costimulatory receptor expressed on T-cells, interacts with ligands on activated APCs (CD80 or CD86), resulting in multiplication of T-cells [6].

T-cells express both CTLA-4 and PD-1 immune checkpoints. Following T-cell activation, CTLA-4 is externalized from intracellular lysosomes in T-cells and binds with CD80 or CD86 (previously known as B7) on APCs, competitively blocking binding to CD28. This process results in downregulation of T-cell-mediated immune response and promotion of immune tolerance [7], which prevents autoimmunity [6]. CTLA-4 appears to play a role in the earlier stages of the immune response, particularly in lymph nodes [8]. CTLA-4 inhibitors oppose the interaction between CTLA-4 and the described ligands, thereby preventing attenuation of immune response [2,3].

Activated T-cells, B-cells, and monocytes also express PD-1, an immune checkpoint which binds to ligands (PD-L1, PD-L2) expressed on APCs, triggering an apoptotic state known as T-cell exhaustion which suppresses immune response. Upregulation of PD-L1 expression may occur in some cancer cells, thus allowing these malignancies to evade the immune system [6]. PD-1 and PD-L1 inhibitors block the interaction between PD-1 and these ligands, such that T-cell inhibitory signaling is halted, resulting in persistent immune system activation [2]. This inhibition of immune effector cell response is most prevalent at the peripheral level and within the localized tumor environment [3]. Both CTLA-4 and PD-1, which are predominantly expressed in immune cells, are expressed in various human tissues, including the pituitary gland [2].

LAG-3 is another immune checkpoint expressed on a variety of immune modulating cells, including activated T-cells, B-cells, NK cells, and dendritic cells. LAG-3 inhibits immune response by binding to several ligands, such as MHC II on APCs, thus preventing interaction between CD4 and MHC II [3]. The LAG-3 inhibitor, relatlimab, maintains the activity of immune cells by preventing expression of the LAG-3 gene [2].

## 2. Animal Models of Autoimmune Hypophysitis

Previously, autoimmune hypophysitis was considered a rare disease, and at one point, the pituitary gland was thought to be resistant to the experimental induction of disease. As summarized by Caturegli et al. [9], several prior attempts of animal models of autoimmune hypophysitis using rodents, rabbits, and rhesus monkey by immunization with pituitary tissues or extracts, even with Freund’s adjuvant, were unsuccessful in recapitulating all the pathological changes observed in autoimmune hypophysitis; however, some of these approaches were able to induce antibodies against the pituitary gland.

In 2008, a new mouse model of experimental autoimmune hypophysitis was established by immunizing female SJL/J mice with mouse pituitary extracts that closely mimicked the human pathology, including pituitary enlargement with compressive symptoms in the initial stage, adrenal insufficiency and pituitary antibodies in the florid phase, followed by pituitary atrophy and hypothyroidism in the late phase [10]. By tracking the changes in the pituitary volume of these mice using serial MRIs up to 300 days after immunization, the authors were able to demonstrate the evolution of autoimmune hypophysitis, including pituitary atrophy from apoplexy in selected animals [11]. Moreover, suppression of hypophysitis was observed when the tumor necrosis factor (TNF)-α pathway was blocked locally, at the site of immunization, and not systemically, by the administration of emulsified placental proteins, providing evidence that the placenta contributes to the immune tolerance of pregnancy by locally inhibiting the TNF-α pathway, reproducing the relationship between autoimmune hypophysitis and pregnancy [12].

Several additional observations using this animal model together with corroborations in the pathological findings in humans led to improved understanding of the pathogenesis of autoimmune hypophysitis. This included the observation of in situ activation of pituitary-infiltrating T-lymphocytes with the CD4+ T-lymphocytes representing the main immune cell population and the contribution of the Th17 subset to the pathology of autoimmune hypophysitis [13,14]. More recently, the interleukin-1 receptor-associated kinase 1 (IRAK1) was found to be upregulated in the pituitaries of mice that developed experimental autoimmune hypophysitis [15]. Moreover, treatment with rosoxacin, a quinolone derivative antibiotic and a specific inhibitor for IRAK1, led to suppression of autoimmune hypophysitis with reduced production of autoantigen-specific antibodies, cytokines, and chemokines that may dampen T-cell differentiation or recruitment to the pituitary [15]. Whether rosoxacin or similar drugs may play a role in the management of autoimmune hypophysitis has not been assessed.

IIH is now the most commonly recognized form of hypophysitis. To improve the understanding of IIH, an animal model was established by repeated injections of a CTLA-4 blocking antibody into SJL/J or C57BL/6J mice, which led to the development of lymphocytic infiltration of the pituitary gland and circulating pituitary antibodies [16]. mRNA and protein expressions of CTLA-4 were demonstrated in a subset of prolactin- and thyrotropin-secreting cells, and these cells became the site of complement activation and an inflammatory cascade similar to that seen in type II hypersensitivity [16]. Moreover, in an autopsy series of six cancer patients treated with CTLA-4 blockade, one had clinical and pathologic evidence of hypophysitis, while a second one had mild lymphocytic infiltration in the pituitary gland without clinical signs of hypophysitis [17]. Although CTLA-4 expression was present in all patients at different levels, the highest expression was noted in the patient with hypophysitis, and this was associated with T-cell infiltration and IgG-dependent complement fixation and phagocytosis, resulting in an extensive destruction of the adenohypophyseal architecture [17]. Additional autopsy studies of six patients with IIH showed that while patients treated with ICI monotherapy developed T-cell predominant lymphocytic infiltrates, those receiving additional therapies demonstrated an increase in B- and T-lymphocytes, with the dominant inflammatory population being type 2 macrophages, and CD25+ T-regs being sparse or absent [18].

Another subtype of autoimmune hypophysitis is lymphocytic infundibulo-neurohypophysitis (LINH), which is characterized by infiltration of the neurohypophysis and/or the hypothalamic infundibulum, causing arginine vasopressin (AVP) deficiency. Based on a small series of patients, rabphilin-3A has been identified as a major autoantigen in LINH, which has led to the recommendation to use autoantibodies to rabphilin-3A as a biomarker for the diagnosis of LINH, and in the differential diagnosis in patients with AVP deficiency [19,20]. Moreover, this was confirmed in an experimental model of LINH. Immunization of female SJL/J mice with rabphilin-3A led to lymphocytic infiltration in the neurohypophysis and supraoptic nucleus after 1 month, and an increase in the volume of hypotonic urine as compared with control mice [21]. Although administration of a cocktail of monoclonal anti-rabphilin-3A antibodies did not induce neurohypophysitis, administration of abatacept, a chimeric protein that suppresses T-cell activation, decreased the number of T-cells specific for rabphilin-3A in peripheral blood mononuclear cells and ameliorated lymphocytic infiltration of CD3+ T-cells in the neurohypophysis [21].

## 3. Immune Checkpoint Inhibitor-Induced Hypophysitis

Autoimmune hypophysitis used to be considered a rare disease; however, the use of immunotherapy in cancer therapy has led to recognition of the diagnostic entity of IIH. In this section, we will review the epidemiology, pathophysiology, clinical presentation, diagnosis, predictors of IIH, and management using a clinical case to illustrate this clinical entity.

### 3.1. Epidemiology of IIH

Endocrine immune-related adverse events (irAEs) may occur in as many as 40% of patients receiving ICI therapy [22]. There exists a positive correlation between the development of irAEs and treatment response. Melanoma patients who develop a hypophysitis on ipilimumab have a higher survival rate than those patients who do not [22]. Hypophysitis occurs in 1.8% to 17% of patients receiving ipilimumab, either alone or in combination with a PD-1/PD-L1 inhibitor, while the incidence of hypophysitis in the setting of PD-1/PD-L1 monotherapy is reported to range between 0.5% and 1.0% [23]. Although LAG-3 inhibitors have not been adequately studied with respect to hypophysitis risk, the addition of a LAG-3 inhibitor to a PD-1 inhibitor appears to raise the incidence of hypophysitis from 0.8% to 2.5% [3,23].

IIH is more common in males over the age of 60 [6], while primary hypophysitis predominately affects women, who also have a lower mean age at diagnosis of 45 years [24]. Pre-existing autoimmunity occurs at rates just slightly higher than baseline in patients who develop IIH [24]. There also appear to be gender-specific differences in presentation, with males developing impairment of multiple axes and experiencing secondary hypothyroidism and hypogonadism more often than females. In patients who develop a hypophysitis, the thyroid axis is affected in 66% of males vs. 35% in females, while the gonadal axis is involved in 66% of males and 21% of females. In a multicenter retrospective study, additional significant differences between primary hypophysitis and IIH were identified, including a higher rate of AVP deficiency (previously known as diabetes insipidus) in patients with primary hypophysitis when compared to IIH (38% vs. 4%) but a higher incidence of hypoprolactinemia with IIH (15% vs. 3%) [24].

### 3.2. Pathophysiology of IIH

Although the exact mechanisms by which ICIs cause hypophysitis remain unclear, current evidence supports the involvement of several potential processes [7]. The underlying pathophysiology may vary based on ICI class [3]. It is postulated that CTLA-4 inhibitors may directly attack anterior pituitary cells, while PD-1/PD-L1 inhibitors may launch a more general immune response, thus damaging pituitary tissues through a more indirect process [3].

Studies have shown that CTLA-4 expression varies amongst individuals and that those having greater pituitary expression of CTLA-4 may be more susceptible to the development of IIH [6,7]. CTLA-4 inhibitor-mediated hypophysitis likely involves a type II hypersensitivity reaction, followed by a type IV hypersensitivity reaction. In a type II (IgG-dependent) hypersensitivity reaction, the antibody–antigen complex activates the classic complement cascade, phagocytes, and B lymphocytes, thereby enhancing the autoimmune response and leading to destruction of pituitary cells, a process similar to that observed in the setting of autoimmune diseases [7]. Occurring later in the course of the autoimmune process is a type IV (T-cell-dependent) hypersensitivity reaction, in which CTLA-4 inhibitors induce autoreactive T-cells as part of a T-cell-mediated cytotoxicity (antibody-dependent cellular cytotoxicy, ADCC) [2,6]. Two autoantibodies have been identified in patients with IIH, including guanine nucleotide-binding protein G (olf) subunit alpha (anti-GNAL) and integral membrane protein 2B (anti-ITM2B Abs) [25].

PD-1 and PD-L1 inhibitor-mediated hypophysitis have a less clear mechanism. It is suspected that these ICIs stimulate the production of pituitary autoantibodies (IgG1 and IgG4) and may resemble a type of paraneoplastic syndrome [2]. Paraneoplastic autoimmune hypophysitis has been described as a possible cause for isolated ACTH deficiency. Ectopic expression of proopiomelanocortin (POMC) has been identified on tumor cells of various primary malignancies. The presence of POMC may then induce the secretion of anti-POMC antibodies, culminating in the autoimmune destruction of pituitary corticotrophs [26]. Additionally, it has also been shown that some malignancies, such as thymomas, ectopically express Pit-1, a necessary transcription factor in the process of differentiation of somatotrophs, lactotrophs, and thyrotrophs in the anterior pituitary. The development of anti-Pit-1 antibodies may lead to damage of these Pit-1-expressing pituitary cells, resulting in selective pituitary hormonal deficiencies in these patients [26,27]. Indeed, in 2024, two cases of anti-PIT-1 hypophysitis were reported after treatment with PD-1 inhibitors [28]. Additional cases of ICI-induced paraneoplastic pituitary hormone deficiency likely will enhance our understanding of the underlying mechanism.

### 3.3. Clinical Case of IIH

A 71-year-old female presented to the Oncology clinic with a diagnosis of locally invasive sinonasal malignant melanoma. Her pre-cancer medical history was significant only for hypertension, treated with candesartan, hydrochlorothiazide, and metoprolol. Following endoscopic resection of the tumor, she was started on combined CTLA-4/PD-1 ICI therapy, with ipilimumab and nivolumab, with plans to complete restaging imaging after 4 cycles of treatment. Following the first cycle of treatment, she developed a Grade 1 thyrotoxicosis and Grade 2 hepatitis. Repeat thyroid function tests 6 weeks later were consistent with hypothyroidism, presumably post-thyroiditis, and she was started on a full replacement dose of levothyroxine. The hepatitis resolved without intervention. She completed three cycles of ipilimumab/nivolumab therapy and was noted to have had an excellent response to treatment based on restaging CT scans. However, a CT head completed for restaging revealed an interval increase in size and heterogeneity of the pituitary gland, with the differential including IIH and the less likely occurrence of pituitary metastases. At the time of this imaging, the patient was asymptomatic, with only mild fatigue being present. A random cortisol level of 375 nmol/L (85–620 nmol/L), drawn 9 days post-CT head, was normal. However, more than 1 month following this initial imaging and laboratory testing, she developed nausea, vomiting, anorexia, and profound fatigue. Subsequent repeat laboratory evaluation revealed a low ACTH of 0.5 pmol/L (1.6–13.9 pmol/L) and low a.m. cortisol of 36 nmol/L (120–620 nmol/L), consistent with a diagnosis of central adrenal insufficiency. A full pituitary panel of blood work ruled out any additional pituitary hormonal deficiencies. Thyroid hormone profile was normal and not suggestive of a central hypothyroidism superimposed on her primary hypothyroidism. TSH was 1.44 mIU/L (0.20–4.00 mU/L), free T4 17.8 pmol/L (9.0–23.0 pmol/L), and free T3 4.2 pmol/L (3.5–6.5 pmol/L). Furthermore, her suppressed estradiol of <30 pmol/L was appropriately associated with gonadotropins elevated into the post-menopausal range.

The patient was started on hydrocortisone 10 mg three times daily, which was then reduced to a more physiologic replacement dose of 10 mg in the morning and 5 mg in the evening, on which she was adequately replaced. She also remained euthyroid on a levothyroxine dose of 100 mcg daily but unfortunately passed away a couple of years following her initial diagnosis after a recurrence of malignant melanoma.

### 3.4. Clinical Presentation of IIH

When present, IIH-related symptoms are due to either hypopituitarism or mass effect from significant pituitary enlargement. Headaches and visual field deficits may be present in patients with the latter [29]. Mass effect symptoms occur more frequently with CTLA-4 inhibitors than with PD-1/PD-L1 inhibitors but are generally uncommon in the setting of ICIs [29]. However, should they be present, MR imaging of the pituitary gland is indicated. While CTLA-4 inhibitors tend to affect multiple pituitary axes, PD-1/PD-L1 more commonly cause isolated ACTH deficiency. ICI class-specific differences are also evident on pituitary MRI, with CTLA-4 inhibitor-associated hypophysitis more commonly being associated with pituitary enlargement, homogeneous enhancement, and stalk thickening, whereas PD-1/PD-L1 inhibitors rarely demonstrate radiologic pituitary changes [3,29,30]. When MRI findings of hypophysitis are present, they typically precede onset of symptoms and biochemical findings of hypopituitarism and are also transient, such that resolution occurs over a period of weeks. The episode of hypophysitis may culminate in the development of an empty sella in some cases [3].

Reported frequencies of IIH are variable and dependent on the types of ICIs used, as well as monotherapy versus dual therapy. According to a retrospective analysis using the FDA Adverse Event Reporting System (FAERS), the frequency of ICI-induced hypophysitis varied by treatment type: 0.80% for PD-1 inhibitors, 0.47% for PD-L1 inhibitors, 1.79% for CTLA-4 inhibitors, and 4.06% for combination therapy (PD-1/PD-L1 + CTLA-4 inhibitors). Combination therapy was associated with the highest reported risk compared to monotherapies (*p* < 0.001), and CTLA-4 inhibitors presented with a significantly higher risk than anti-PD-1 or anti-PD-L1 monotherapies (*p* < 0.001) [31]. A recent analysis of 159 patients who received relatlimab–nivolumab combination therapy reported that the combination therapy significantly increased the incidence of adrenal insufficiency, likely due to hypophysitis, when compared to nivolumab monotherapy, with 7.6% of patients on combination therapy developing the condition versus 0.8% of those on nivolumab alone [32]. Moreover, in a recent autopsy series of 42 patients treated with ICIs, hypophysitis was clinically unsuspected in 8 of 12 (66.7%) cases [33]. Therefore, a strategy to recognize IIH in particular patients with cortisol deficiency is required.

Symptoms of IIH related to hypopituitarism are variable and often nonspecific and may include fatigue, nausea, vomiting, anorexia, hypotension, orthostatic dizziness, and weight loss [34]. Symptoms may overlap with those related to malignancy or concurrent non-ICI therapies [22]. When signs and symptoms of AVP deficiency, such as polyuria and polydipsia, are present, an MRI is indicated to assess for possible pituitary metastases, given the rarity of ICI-induced vasopressin deficiency [34]. IIH occurs at a median time of 8–12 weeks when due to CTLA-4 inhibitor use and at a median of 24–26 weeks with PD-1/PD-L1 inhibitor use, although earlier development, at a median of 13.5 weeks, has also been reported [29,34].

### 3.5. Diagnosis of IIH

The most commonly affected pituitary axes, in descending order, are the adrenal, thyroid, and gonadal axes [3]. Because the presence of adrenal insufficiency, in particular, may be life-threatening, prompt recognition and management are crucial. Because IIH most commonly occurs within the first 6 months of therapy, monthly monitoring of TSH, free T4, and morning cortisol levels is recommended for 6 months, followed by every 3 months, and then every 6–12 months thereafter [22]. Of note, onset of irAEs, including hypophysitis, may occur up to 6–15 months or more after discontinuation of ICI therapy [27].

Both cortisol and thyroid hormone deficiencies may present with hyponatremia [3]. Therefore, a diagnosis of adrenal insufficiency and/or hypothyroidism should be entertained in patients who have low serum sodium levels. Additionally, eosinophilia is frequently observed in patients with adrenal insufficiency [35]. In those who develop adrenal insufficiency, it is important to assess an ACTH level to rule out the rare occurrence of primary adrenal insufficiency [27]. It is important to recognize that an ACTH stimulation test may be falsely normal in the acute setting [27], as it may take 6–12 weeks for the adrenal glands to atrophy from lack of ACTH stimulation [22]; therefore, an ACTH stimulation test is not a suitable diagnostic tool for identifying central adrenal insufficiency associated with IIH.

Hypogonadotropic hypogonadism may present with low energy, loss of muscle mass, reduced libido, and erectile dysfunction in males, while females may experience menstrual irregularities or amenorrhea [23]. Of note, acute or chronic illness may suppress the hypothalamic–pituitary–gonadal axis and have a similar clinical and biochemical presentation. Because thyrotropin and gonadotropin deficiencies may resolve, it is acceptable to postpone initiation of thyroid and/or sex hormone replacement if the abnormality is mild or improving [36]. Recovery of central hypothyroidism occurs in up to 85% of patients, while recovery of central hypogonadism occurs in up to 83% of individuals [34]. Neuroimaging differences also exist between IIH and primary hypophysitis, with the latter more frequently displaying MRI findings of pituitary stalk thickening (56% vs. 27%). Furthermore, abnormal findings, such as stalk thickening, may be transient and observed for a shorter duration in IHH compared to primary hypophysitis [24].

Halting ICI therapy after the development of a hypophysitis has not been shown to reverse hypopituitarism or improve outcomes, such that routine discontinuation of ICI treatment is not recommended; however, temporarily holding therapy in patients with grades 3 and 4 toxicity, until their condition improves, is suggested in some guidelines [34]. Of note, the onset of IIH is associated with a more favorable prognosis, potentially indicating a therapeutic effect on cancer [22,37].

CTLA-4 inhibitor-induced hypophysitis affects several hormonal axes, occurs earlier in the course of treatment, and is dose-dependent. Several studies have reported higher rates of IIH in patients receiving ipilimumab doses of 10 mg/kg, rather than 3 mg/kg [22].

### 3.6. Predictors of IIH

In a case-controlled study of patients treated with ICIs, all of whom had negative anti-pituitary antibodies at baseline, Kobayashi et al. [38] showed that the development of anti-pituitary antibodies was associated with the onset of hypophysitis. Additionally, they also identified specific HLA alleles increasing susceptibility to either IIH (HLA-Cw12 and HLA-DR15) or ICI-induced isolated ACTH deficiency (HLA-Cw12, HLA-DR15, HLA-DQ7, and HLA-DPw9) [38]. Furthermore, melanoma patients who develop IIH may demonstrate a rapid drop in serum sodium and an absolute increase in eosinophil and lymphocyte counts, and so these additional biomarkers may suggest the development of IIH [39]. A reduction in TSH from baseline levels [40], or a reduction in free T4 and TSH index (a free T4 adjusted TSH) [41], may signal the initial stages of a hypophysitis, with the caveat that similar biochemical changes may be observed in the setting of nonthyroidal illness and/or therapy with supraphysiologic doses of glucocorticoids.

The incidental identification of hypophysitis on MRI, in individuals who are asymptomatic and have no biochemical evidence of hypopituitarism, may portend the development of pituitary insufficiency. In these individuals, surveillance of morning serum cortisol levels is recommended for a period of at least one month, to monitor for the possible evolution of adrenal insufficiency [22].

### 3.7. Management of IIH

In patients with IIH associated with ACTH deficiency, initiation of glucocorticoid replacement is mandatory and considered life-sustaining. In most cases, physiologic dosing is advised, for example, with hydrocortisone 15–20 mg daily, divided into 2–3 daily doses. High-dose glucocorticoids, which are the mainstay of therapy for non-endocrine irAEs, do not improve pituitary function and should not be routinely prescribed [34]; in fact, supraphysiologically dosed glucocorticoids may reduce survival in melanoma patients with ipilimumab-induced hypophysitis, as was shown in one retrospective review [42]. Additionally, high-dose steroids may either cause or exacerbate osteoporosis and type 2 diabetes mellitus [34]. Treatment with high-dose prednisone (1–2 mg/kg/day) is primarily indicated in the minority of patients who develop compressive symptoms from pituitary gland enlargement. When concurrent central hypothyroidism is present, it is important to start glucocorticoid therapy prior to thyroid hormone replacement in order to avoid precipitating an adrenal crisis [23]. Patients with persistent hypogonadotropic hypogonadism 3–6 months following a diagnosis of IIH may be candidates for testosterone or estrogen replacement [6], and the administration of estrogen or testosterone replacement therapy should be carefully evaluated based on the patient’s overall health status and age. Patients who develop growth hormone deficiency as a consequence of IIH should not be treated with growth hormone, which would be contraindicated in the setting of an active malignancy [2].

## 4. Immune Microenvironment and Pituitary Neuroendocrine Tumor (PitNET) with PDL-1 as a Biomarker

Although IIH is not rare in patients on ICI therapy, this treatment modality has only been used in a small number of patients with aggressive or metastatic PitNETs. In this section, we will summarize the role of the immune microenvironment on the pathogenesis, behavior, and management of PitNETs, outline the use of PDL-1 as a biomarker, and describe treatment outcomes of ICI therapy.

### 4.1. Immune Microenvironment of PitNETs

**Figure 1:**
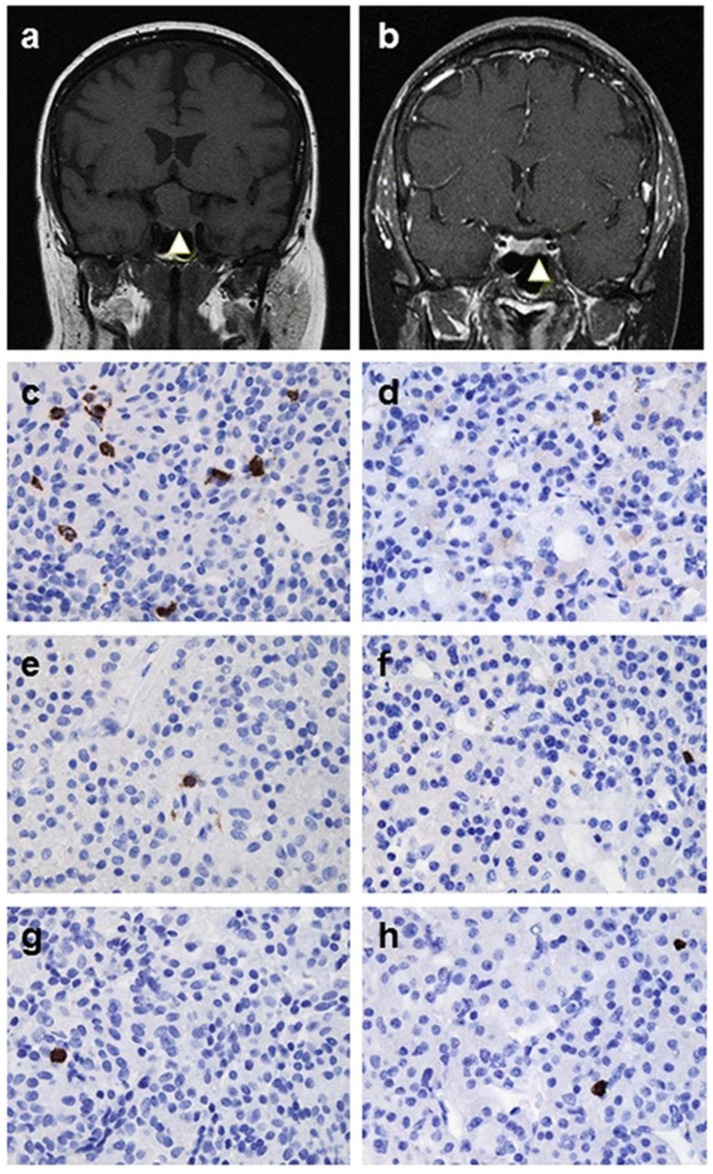
Immune cell infiltrates in pituitary macroadenoma and microadenomas. A T1-weighted MR image shows a macroadenoma ((**a**); null cell adenoma, arrow), which photomicrograph demonstrates scattered CD68+ macrophages (**c**), rare CD4+ cells (**e**), and rare CD8+ cells (**g**). In contrast, a Gadolinium-enhanced T1-weighted MR image exhibits a microadenoma ((**b**); ACTH adenoma, arrow) which photomicrograph demonstrates sparse CD68+ macrophages (**d**), rare CD4+ cells (**f**), and rare CD8+ cells (**h**). Original magnifications ×400 (**c**–**h**). Reproduced with permission from [44] Springer Nature: Lu JQ/Chik CL, et al. Endocrine Pathology 2015;26:263-72.

PitNETs are a diverse group of tumors arising from the pituitary gland. The immune microenvironment of these tumors is complex and plays a crucial role in their behavior and progression. It is now recognized that the immune system plays a crucial role in the development and progression of various benign and malignant neoplasms [43]. Actions of the immune system include promoting an inflammatory microenvironment, which contributes to genomic instability and epigenetic modifications, resulting in increased cell proliferation, angiogenesis, and activation of anti-apoptotic pathways [43]. A significant proportion of PitNETs exhibit varying degrees and types of immune cell infiltration [44,45,46,47], as demonstrated in a recent case of a 2.6 cm sparsely granulated somatotroph PitNET, a relatively aggressive subtype of PitNET (Figure 1).

Previously, we have found that the number of CD68+ macrophages was positively correlated with aggressive behaviors of PitNETs, including tumor sizes and Knosp classification grades [44]. PIT1-lineage PitNETs exhibit significant M2 macrophage infiltration, which is associated with tumor aggressiveness [48]. This overexpression correlates with larger tumor volumes and invasive behavior, such as cavernous sinus invasion. Additionally, B-lymphocytes and natural killer (NK) cells are known to infiltrate PitNETs [49]. Immune cells within the PitNET microenvironment display diverse phenotypes and secrete various cytokines and chemokines, including pro-inflammatory and immunomodulatory mediators [49]. These factors are implicated in tumor progression, angiogenesis, and hormone secretion, thereby shaping the tumor’s biological behavior and potentially affecting its clinical presentation and therapeutic responsiveness. In functioning PitNETs, the extent of macrophage infiltration is three to four times greater than in non-tumoral pituitary tissue [50]. Based on their immunological profile and the expression of different immune checkpoint molecules, PitNETs have been classified into various categories, including tumors sensitive to anti-CTLA-4 monoclonal antibodies, tumors sensitive to anti-PD-L1 monoclonal antibodies, and tumors resistant to both anti-CTLA-4 and anti-PD-L1 antibodies [6,45,51].

### 4.2. PD-L1 as a Biomarker

PD-L1 is a transmembrane protein that plays a crucial role in immune system regulation, particularly in the context of cancer. PD-L1 is expressed on various cell types, including macrophages, non-lymphoid tissues such as the heart and lungs, and cancer cells. By interacting with its receptor, PD-1, on T-cells, PD-L1 inhibits T-cell activity, thus allowing cancer cells to evade immune detection and destruction [48]. This immune evasion mechanism is responsible for the progression of various cancers, making PD-L1 a significant biomarker and therapeutic target in oncology.

The therapeutic blockade of the PD-1/PD-L1 pathway using monoclonal antibodies, such as nivolumab, pembrolizumab, and atezolizumab, has shown substantial efficacy in enhancing anti-tumor immunity [52]. These inhibitors can restore T-cell activity, leading to improvement in the ability of the immune system to target and eliminate cancer cells. Higher PD-L1 expression levels are often associated with a more effective response to these immunotherapies, highlighting the importance of PD-L1 as a predictive biomarker.

Recent studies have highlighted the role of PD-L1 in PitNETs. PD-L1 expression has been associated with more aggressive tumor behavior in various solid tumors, such as large-cell NET carcinomas at various sites [53,54,55,56], and its expression in PitNETs has been linked to higher proliferation rates and poorer clinical outcomes [57]. The distinct immune profile of PitNETs, especially the high PD-L1 expression, suggests that ICIs targeting PD-L1 could be a promising therapeutic approach for aggressive and refractory PitNETs.

### 4.3. A Review of Immunotherapy in the Management of Aggressive and Metastatic PitNETs

We reviewed the literature on the use of ICIs in patients with aggressive and refractory PitNETs until 31 March 2025 and found 40 cases (Table 1) [58,59,60,61,62,63,64,65,66,67,68,69,70,71,72], including 4 cases which were part of a phase 2 clinical trial [62], 15 cases from a French multicentric retrospective cohort study [66], and 9 cases from another phase 2 clinical trial [72].

Together, 25 patients, including 16 with metastasis, had corticotroph PitNETs, and 13 patients, including 6 with metastasis, had lactotroph PitNETs. Of the remaining two patients, one had a metastatic PitNET of Pit-1 lineage, and a second had a somatotroph PitNET. All patients had had one or more pituitary surgeries, all but one patient had had radiation surgery, and all but one patient had had prior treatment with temozolomide. Additional multimodal therapies had also been used, including medical therapies, other chemotherapeutic agents, and surgeries for resection of metastasis and bilateral adrenalectomy (the first 31 cases were reviewed by Lopes-Pinto et al.) [43,72]. Whereas 31 patients were treated with dual therapy with nivolumab and ipilimumab, 8 patients were treated with pembrolizumab. One patient started with nivolumab monotherapy, and another patient was on an anti-PD-1 inhibitor. The response duration was difficult to assess, as the treatment protocols were heterogeneous, ranging from 2 to 17 cycles of therapy over a period of 2 to 42 months.

Response rates (complete + partial response + stable disease) differed depending on whether the calculations were based on the initial response or the final response after treatment. Based on imaging response, the initial response rate was 60% (complete response in 1 patient, partial response in 8 patients, stable disease in 15 patients), and progressive disease was observed in 14 patients. Three patients displayed dissociated responses between the tumor and the metastasis. By the end of the treatment duration, the response rate was 38%, as 9 additional patients had progressive disease, including 1 with an initial partial response, 7 with initial stable disease, and 1 with an initial dissociated response. Furthermore, 1 additional patient with an initial partial response ultimately demonstrated a dissociated response. Among the 24 functioning PitNETs, hormonal responses were reported in 17 patients, with a response rate of 59%, including 4 with a complete response, 5 with an initial partial response, 1 with stable hormonal levels, and 7 with hormonal progression.

Whether the response rate to ICI therapy is related to the presence of metastasis or the type of PitNET remains unclear. After excluding the 9 patients from the recent cohort study due to unavailability of individual patient responses [72], the initial response rate in the 19 patients with metastatic PitNETs was 53%. This included 6 corticotroph (1 complete response, 5 partial response), 3 lactotroph (2 partial response and 1 stable disease), and 1 Pit-1 lineage (partial response) PitNETs. Two additional patients with corticotroph carcinomas had dissociated response. At the end of the treatment duration, the response rate was 37% (5 corticotroph, 1 lactotroph, and 1 Pit-1 lineage). In comparison, the initial response rate of the 12 patients with aggressive PitNETs was 58%. This included 5 corticotroph PitNETs and 2 lactotroph PitNETs, both with stable disease. At the end of the treatment duration, the response rate was 8%, as only one patient with an aggressive corticotroph PitNET had stable disease. These results are consistent with the conclusion of the French multicentric cohort study that ICIs may represent a good therapeutic option for pituitary carcinomas [66]. However, negative PD-L1 staining and a very low CD8+ T-cell infiltrative in the tumor should not preclude ICI administration in corticotroph carcinomas [66]. In our analysis, among the 24 cases with PD-L1 staining, the two patients with positive PD-L1 staining at 95% (1 with a lactotroph carcinoma and 1 with a Pit-1 lineage carcinoma) both had a partial response (Table 1). A partial response was also observed in 6 patients with negative or <1% PD-L1 staining (4 with corticotroph carcinomas and 2 with lactotroph carcinomas). Taken together, these findings support the inference that negative PD-L1 staining should not preclude ICI treatment of metastatic PitNETs.

With the limited number of cases and the potential for reporting bias, the impact of ICIs on the biological behaviors of PitNETs, including hormone dysregulation, remains to be fully elucidated. Additional clinical trials with standardized protocols are probably necessary to help select patients who are most likely to respond to ICIs and also to ascertain the usefulness of PD-L1 as a target for immunotherapy in refractory cases of PitNETs. The recent identification of mismatch repair deficiency and temozolomide hypermutation as potential biomarkers of response to ICIs based on a biomarker discovery cohort of 13 patients with aggressive or metastatic PitNETs [72] also warrants further evaluation.

## 5. Conclusions

This review synthesizes current knowledge on IIH and the immunomodulatory landscape of PitNETs. We emphasize the clinical recognition of IIH and the critical need for prompt management. As well, we explore the role of PD-1 and PD-L1 as biomarkers for ICI therapy in aggressive PitNETs. Whereas ICIs offer therapeutic potential in the management of aggressive and metastatic PitNETs, robust clinical trials and translational research are needed to elucidate long-term efficacy and safety and to ensure appropriate patient selection and optimal timing of immunotherapy in the management algorithm of these complex patients.

## Figures and Tables

**Table 1:** Summary of the 40 patients with aggressive or metastatic PitNETs treated with immunotherapy.

Ref	Age ^a^	Sex	PitNET	Function	Carcinoma	PD-L1	Treatment	Imaging Response	Hormone Response
[58]	35	F	ACTH	ACTH	Yes	<1%	Dual to Niv	PR → DPR	PR → PD → SD
[59]	47	M	ACTH	Silent to ACTH	No	Neg	Pembro	PD	PD
[60,66]	60	F	ACTH	ACTH	Yes	Neg	Dual to Nivo	DPR → PD	PD
[60,66]	68	M	PRL	PRL	No	Neg	Dual	PD	PR → PD
[61]	72	F	PRL	Silent	Yes	<1%	Dual to Nivo to Dual	PR → PD	Silent tumor
[62]	30s	M	ACTH	ACTH	Yes	Neg	Pembro	PR	CR
[62]	20s	F	ACTH	ACTH	Yes	Neg	Pembro	PR	PD → PR
[62]	Teens	M	ACTH	Silent	Yes	Neg	Pembro	PD	Silent tumor
[62]	50s	F	PRL	PRL	Yes	Neg	Pembro	SD	PD
[63]	48	M	ACTH	ACTH	Yes	NA	Dual to Niv	CR	PR
[64]	57	M	ACTH	Silent	Yes	NA	Dual	PD	Silent tumor
[65]	NR	NA	ACTH	ACTH	No	15%	ICI not specified	PD	NA
[65]	NR	NA	ACTH	ACTH	Yes	NA	Dual	PD	NA
[65]	NR	NA	ACTH	ACTH	Yes	NA	Dual	PD	NA
[66]	66	M	ACTH	ACTH	No	NA	Dual	SD → PD	PD
[66]	73	M	PRL	Silent	No	10%	Dual to Nivo	SD → PD	Silent tumor
[66]	73	F	PRL	PRL	No	0%	Dual	SD → PD	PD
[66]	72	F	ACTH	ACTH	No	0%	Dual	SD → PD	PD
[66]	44	M	ACTH	ACTH to Silent	No	0%	Nivo to Ipi	SD	Silent tumor
[66]	31	F	ACTH	ACTH	No	0%	Dual to Nivo	SD → PD	NA
[66]	75	M	PRL	PRL	No	40%	Dual to Nivo	PD	PR → PD
[66]	43	M	ACTH	ACTH	Yes	0%	Dual	PD	NA
[66]	54	F	ACTH	ACTH	No	0%	Dual	SD → PD	SD → PD
[66]	39	M	PRL	PRL	Yes	NA	Dual to Nivo to Ipi	SD → PD	PR → PD
[66]	38	M	ACTH	ACTH	Yes	0%	Dual to Nivo	PR	CR
[66]	52	M	ACTH	ACTH	Yes	0%	Dual to Nivo	DPR	NA
[67]	57	F	Pit-1	Silent	Yes	95%	Pembro	PR	Silent tumor
[66,68]	54	M	PRL	PRL	Yes	95%	Dual to Nivo	PR	CR
[69]	61	M	PRL	PRL	No	Pos	Pembro	PD	NA
[70]	70	F	ACTH	ACTH	Yes	NA	Dual to Nivo	PR	CR
[71]	51	M	ACTH	Silent	Yes	0%	Dual	PD	Silent tumor
[72] ^b^	31–79	M—4 ^c^ F—5	ACTH 4 ^c^ PRL 4 GH 1	Silent/partial 4, ACTH 1 ^c^	ACTH 2 PRL 2	NA	Dual to Nivo	SD—6 PD—3	>50% ↓, none

^a^ age at treatment with immunotherapy; ^b^ a cohort of 10 patients; ^c^ 1 patient withdrew before treatment; ACTH, adrenocorticotropic hormone, CR, complete response; DPR, dissociated partial response between the pituitary tumor and metastasis; Dual, ipilimumab and nivolumab; F, female; ICI, immune checkpoint inhibitor; Ipi, ipilimumab; M, male; NA, not available; Nivo, nivolumab; PD, progressive disease; PD-L1, programmed cell death ligand-1 status; Pembro, pembrolizumab; PR, partial response; PRL, prolactin; SD, stable disease, ↓, reduction.

## Data Availability

Anonymized relevant clinical data and representative immunostaining images are presented within the manuscript. Further inquiries regarding specific data points can be directed to the corresponding authors.

## Abbreviations

The following abbreviations are used in this manuscript:

ICIs	immune checkpoint inhibitors
IIH	ICI-induced hypophysitis
PitNETs	pituitary neuroendocrine tumors
PDL-1	programmed cell death ligand 1
FDA	Food and Drug Administration
CTLA-4	cytotoxic T-lymphocyte-associated antigen 4
LAG-3	lymphocyte-activating gene-3
PD-1	programmed cell death protein 1
APCs	antigen presenting cells
MHC	major histocompatibility complex
TCR	T-cell receptor
IRAK1	interleukin-1 receptor-associated kinase 1
LINH	lymphocytic infundibulo-neurohypophysitis
AVP	arginine vasopressin
irAEs	immune-related adverse events
ADCC	antibody-dependent cellular cytotoxicity
GNAL	guanine nucleotide-binding protein G (olf) subunit alpha
ITM2B	integral membrane protein 2B
ACTH	adrenocorticotropic hormone
POMC	Proopiomelanocortin
NK	natural killer
